# Phylogenetic analysis and structural prediction reveal the potential functional diversity between green algae SWEET transporters

**DOI:** 10.3389/fpls.2022.960133

**Published:** 2022-09-15

**Authors:** Jack Fleet, Mujtaba Ansari, Jon K. Pittman

**Affiliations:** ^1^Department of Earth and Environmental Sciences, Faculty of Science and Engineering, School of Natural Sciences, The University of Manchester, Manchester, United Kingdom; ^2^School of Biological Sciences, Faculty of Biology, Medicine and Health, The University of Manchester, Manchester, United Kingdom

**Keywords:** Chlorophyta, evolution, green algae, phylogeny, protein structure, sugar transport, SWEET

## Abstract

Sugar-Will-Eventually-be-Exported-Transporters (SWEETs) are an important family of sugar transporters that appear to be ubiquitous in all organisms. Recent research has determined the structure of SWEETs in higher plants, identified specific residues required for monosaccharide or disaccharide transport, and begun to understand the specific functions of individual plant SWEET proteins. However, in green algae (Chlorophyta) these transporters are poorly characterised. This study identified SWEET proteins from across representative Chlorophyta with the aim to characterise their phylogenetic relationships and perform protein structure modelling in order to inform functional prediction. The algal genomes analysed encoded between one and six SWEET proteins, which is much less than a typical higher plant. Phylogenetic analysis identified distinct clusters of over 70 SWEET protein sequences, taken from almost 30 algal genomes. These clusters remain separate from representative higher or non-vascular plant SWEETs, but are close to fungi SWEETs. Subcellular localisation predictions and analysis of conserved amino acid residues revealed variation between SWEET proteins of different clusters, suggesting different functionality. These findings also showed conservation of key residues at the substrate-binding site, indicating a similar mechanism of substrate selectivity and transport to previously characterised higher plant monosaccharide-transporting SWEET proteins. Future work is now required to confirm the predicted sugar transport specificity and determine the functional role of these algal SWEET proteins.

## Introduction

Sugars are essential components of carbon metabolism, and are a key source of energy for growth and development in all organisms. Photosynthetic organisms including plants and algae are valuable sources of sugars both for nutritional uses but also for other industrial applications, such as for fermentation to produce biofuels (Bhaumik and Dhepe, 2016; Figueroa-Torres et al., 2020). The ability of many algae strains (particularly unicellular microalgae) to achieve fast growth and high biomass yield, and with the potential for cultivation under conditions that will not compete with agriculture, allows for a more scalable and sustainable source of fermentable sugars (Chen et al., 2013). It is therefore important to understand the molecular mechanisms of sugar metabolism and transport in algae in order to maximise their potential as a renewable fuel feedstock (Johnson and Alric, 2013), as well as to enhance our fundamental understanding of carbohydrate metabolism processes in these organisms.

In higher plants, there are several classes of sugar transporter that play crucial roles in sugar partitioning and carbohydrate metabolism, which include the more recently discovered Sugar Will Eventually be Exported Transporter (SWEET) family (Chen et al., 2010; Julius et al., 2017). They are bidirectional uniporters that can allow the movement of sugars across a membrane as determined by the concentration gradient (Xue et al., 2022). The discovery of SWEETs was a notable development, particularly as they were found in all Kingdoms of life, have a broad range of functionality and high level of conservation between species (Jia et al., 2017). As a result, there is significant, ongoing investigation into the evolution of SWEET proteins, their structure, and their functional purpose in organisms. In plants, SWEETs participate in both monosaccharide and disaccharide transport across the plasma membrane and organellar membranes (Chen et al., 2010, 2012) and are shown to have a diverse range of functionality. This includes providing sugars to fungal symbionts or pathogens (Gao et al., 2018; Jeena et al., 2019; Gupta et al., 2021), mediating sucrose efflux from sink tissue cells and vascular pathway cells during phloem loading or unloading (Chen et al., 2012; Lin et al., 2014), or mobilising sugars during plant development (Guan et al., 2008; Shammai et al., 2018). Recent evidence suggest SWEETs may even have the capacity to transport non-sugar substrates. Screening for gibberellin transporters revealed the capacity for low-affinity bidirectional transport by AtSWEET13 and AtSWEET14 (Kanno et al., 2016). Alternatively, analysis of OsSWEET3a revealed dual functionality as both a glucose and gibberellin transporter, mediating phloem loading and early plant development (Morii et al., 2020). Full functionality and diversity of plant SWEET proteins are yet unknown.

Phylogenetic characterisation of SWEETs in angiosperms is based around the nomenclature given to the first identified SWEETs from *Arabidopsis thaliana* (Chen et al., 2010). AtSWEET1 to AtSWEET17 were categorised into four distinct clades: SWEET1–3 (Clade I), SWEET4–8 (Clade II), SWEET9–15 (Clade III), and SWEET16–17 (Clade IV; Eom et al., 2015; Sui et al., 2017). SWEETs in each clade are distinguished in part by their propensity to transport monosaccharides (Clade I, II, IV) or disaccharides (Clade III). Clade I and II SWEETs exclusively transport monosaccharide sugars including glucose and mostly localise to the plasma membrane, although some isoforms show vacuole membrane localisation (Chen et al., 2010, 2015). Preferential disaccharide (mainly sucrose) transport is a characteristic of Clade III SWEETs, which localise to intracellular or plasma membranes (Chen et al., 2012; Yuan and Wang, 2013; Lin et al., 2014; Gao et al., 2018). Clade IV SWEETs are distinguished from the other clades as they exclusively localise to the vacuole and have the capacity to transport several different monosaccharide sugars (Chen et al., 2010; Chardon et al., 2013; Guo et al., 2014). This functional variation between SWEET clades can be attributed to specific amino acids within conserved motifs on SWEET proteins. For example, the addition of a conserved Trp residue in the second transmembrane (TM) helix distinguishes Clades I and II, from Clades III and IV. An additional conserved motif containing the positively charged amino acids His and Arg, distinguishes Clade II SWEETs from Clade I, whereas the conservation of Arg and Trp residues within a Clade III motif distinguishes SWEETs from Clade IV (Jia et al., 2017).

**Figure 1:**
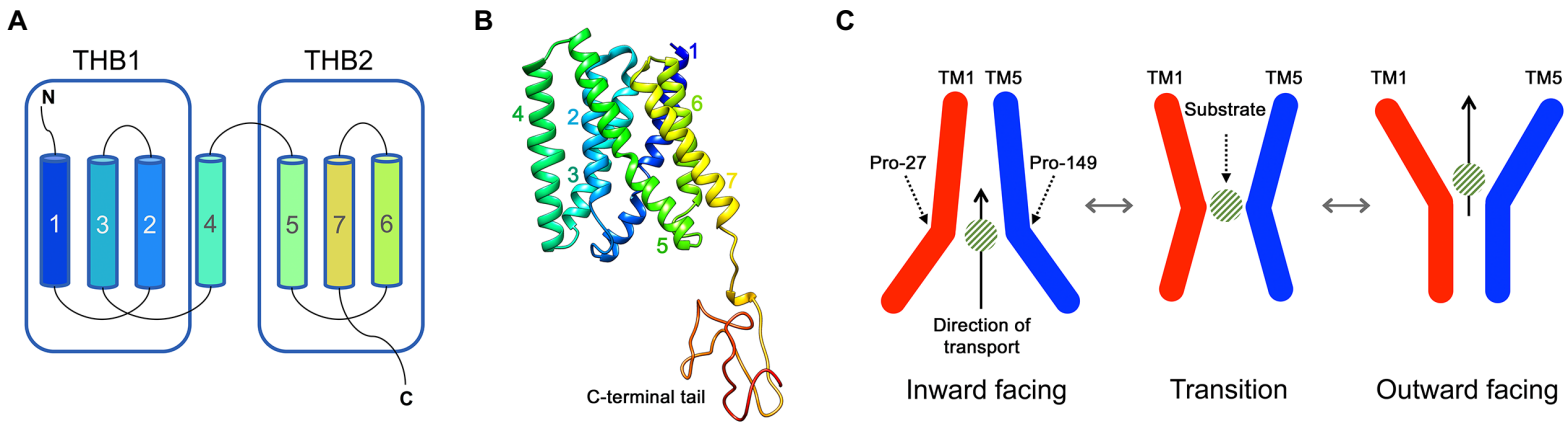
The structure and transport mechanism of SWEET proteins. **(A)** A schematic of a typical eukaryotic SWEET showing the seven transmembrane (TM) helices that are organised into two TM helix bundles (THB). **(B)** The crystal structure of AtSWEET13 with each TM helix numbered. **(C)** The proposed transport mechanism of SWEET proteins. Two Pro residues (such as Pro27 and Pro149, numbered according to AtSWEET13) act as molecular hinges allowing the opening and closing of intra- and extracellular cavities for the translocation of a sugar substrate across a membrane.

SWEETs are characterised by a distinct TM structure: plant SWEETs possess seven TM helices arranged into two TM helix bundles (THB). Each THB contains three TM helices linked by a central TM4 helix (Figure 1A), with this structure referred to as a 3-1-3 formation (Chen et al., 2010). Each THB structure contains a conserved PQ-loop repeat motif (Pfam: PF04193; Xuan et al., 2013). Crystal structures of SWEET proteins from rice (*Oryza sativa*; OsSWEET2b) and *A. thaliana* (AtSWEET13) distinguish the TM helices within the THBs, such that THB1 forms the N-terminal half of the protein arranged in a (1-3-2) + 4 structure, while THB2 forms the C-terminal half arranged in a (5-7-6) formation (Figures 1A,B). Spanning the membrane, TM4 aligns closer to THB1 and covalently fuses THB1 and THB2 asymmetrically, creating a pore for substrate translocation (Tao et al., 2015; Han et al., 2017; Jia et al., 2017). Conformational change mediated by substrate binding drives an alternating access mechanism of passive transport of the substrate through the pore (Figure 1C) with specific residues critical to this mechanism (Latorraca et al., 2017; Selvam et al., 2019). There is also evidence that plant SWEETs can form functional homo- or hetero-oligomers (Xuan et al., 2013; Tao et al., 2015; Han et al., 2017). Prokaryotic SWEET proteins are arranged with a single THB (1-3-2), hence are dubbed SemiSWEETs (Xu et al., 2014), but they can symmetrically dimerise without a TM4 inversion linker to create a pore for the transport of sugars (Xuan et al., 2013). Given the high conservation of the (1-3-2) THB arrangement between SWEETs and SemiSWEETs, it has been hypothesised that eukaryotic SWEETs evolved from a fusion of SemiSWEETs, which have also been identified in eukaryotes including higher plants and algae (Jia et al., 2017).

There has been very limited investigation into the SWEET family in unicellular eukaryotes, and particularly with regard to the various taxonomic groupings of algae. Algae SWEETs are often referred to as “outliers” to contextualise larger angiosperm clades (Eom et al., 2015; Jia et al., 2017;Hu et al., 2018; Li et al., 2018), although there is some evidence that algae SWEETs form a distinct clade to other eukaryote SWEETs alongside fungi-derived SWEETs (Hu et al., 2016; Jia et al., 2017). The evolutionary history of SWEET genes across the algae and plant lineage is not well understood. Whole genome duplication events are likely responsible for the expansion of the SWEET gene family (16–53 per species) in higher plants (Li et al., 2018). In contrast, dispersed duplication as a result of adaptation to environmental changes is a possible explanation for the low number of homologues in algae (Hu et al., 2018; Li et al., 2018). Characterisation of green algae (Chlorophyta) SWEETs is important to both further our understanding of the ancestral origins of higher plant SWEETs, since Chlorophyta SWEETs are closely related to Clade II plant SWEETs (Li et al., 2018), but also to further our understanding of sugar transport within algae themselves. Chlorophyta microalgae represent viable targets for engineered biorefineries for the generation of fermentable sugars, biodiesel lipids and other valuable chemicals (Usher et al., 2014; Banerjee et al., 2021; Figueroa-Torres et al., 2021).

Compared with higher plants, there is a considerable gap in understanding of sugar transport processes in Chlorophyta algae. Hexose uniporter transporters in *Chlorella kessleri* can provide low-affinity, bidirectional glucose transport (Caspari et al., 1994) while transgenic expression of one of these transporters (CkHUP1) into *Chlamydomonas reinhardtii* resulted in improved dark heterotrophic growth on a glucose carbon source and improved the capacity for downstream H_2_ production (Doebbe et al., 2007). Beyond this, there has been little characterisation of the native function of these sugar transporters. Some putative chloroplast-localised transporters, such as a hexose transporter and a triose-phosphate translocator, have been implicated in algal carbohydrate metabolism (Johnson and Alric, 2013; Marchand et al., 2018), while intracellular maltose transport by *C. reinhardtii* MEX1 is important for starch metabolism (Findinier et al., 2017). Furthermore, there is very limited investigation into algal plasma membrane-localised sugar transporters with the capacity for sugar efflux or influx. One study indicated a possible hexose transporter of the green alga *Micractinium conductrix* that could efflux maltose or glucose to support endosymbiotic growth of *Paramecium bursaria*, although the gene encoding this hypothesised transporter was not identified (Arriola et al., 2018). Genomic and proteomic studies have begun to indicate the breadth of transporters present in chlorophytes (Blaby-Haas and Merchant, 2019), although experimental validation and further bioinformatic study of sugar transporters including SWEETs is still lacking at this stage.

This present study has investigated the broad presence of the SWEET transporter family across several green algae, identifying a potential “Chlorophyta clade” of SWEETs external to the known clade structure in higher plants, alongside a comparison with some selected fungal SWEET proteins. Using a combination of phylogenetic and structural analysis, this study has begun to fill the knowledge gap for SWEET proteins in green algae. We examine whether proteins from the same genus or species cluster together or whether there is evidence of similarity of the basis of putative substrate characteristics but independent of taxonomy. Furthermore, we discuss the endogenous functions of these SWEET proteins but also consider how these proteins could be manipulated and exploited for microalgae biotechnological applications.

## Materials and methods

### Identification of chlorophyta SWEETs

The four previously identified but not experimentally characterised *C. reinhardtii* SWEET gene sequences were obtained from *C. reinhardtii* CC-503 MT+ genome (Merchant et al., 2007) assembly and annotation data v.5.6, which was obtained from the DOE Joint Genome Institute (JGI) Phytozome v.13 genome data portal (Goodstein et al., 2011). The *C. reinhardtii* SWEET genome ID numbers were: Cre06.g271800, Cre06.g275000, Cre07.g340700, and Cre10.g421650, named here as CrSWEET1 to 4, respectively. Alongside the *C. reinhardtii* SWEETs, several previously well-characterised higher plant SWEETs from *A. thaliana* and *O. sativa* that represent a cross-section of Clade I to IV SWEETs were used as query sequences in protein homology searches. These included AtSWEET1 (UniProt accession number: Q8L9J7), AtSWEET4 (Q944M5), AtSWEET5 (Q8LBF7), AtSWEET8 (Q8LFH5), AtSWEET13 (Q9FGQ2), OsSWEET2b (Q5N8JI) and OsSWEET11 (Q6YZF3). Using these *C. reinhardtii* and plant amino acid sequences as references, BLASTp searches were carried out in several databases (UniprotKB/SwissProt; UniprotKB Viridiplantae) using different search engines (under E > 0.001): EBI-BLASTp, NCBI-BLASTp, JGI Phytozome-BLASTp (Camacho et al., 2009; Goodstein et al., 2011). Candidate SWEET sequences from Chlorophyta algae were logged and cross-compared to SWEET sequences present in a database of SWEETs across several Kingdoms (including Viridiplantae), as well as those identified by EBI-HMMER protein homology searches (Madeira et al., 2019).

Chlorophyta SWEET proteins were identified from several available genomes from organisms of the order Chlamydomonadales (including five *Chlamydomonas* species: *C. reinhardtii*, *Chlamydomonas debaryana*, *Chlamydomonas eustigma*, *Chlamydomonas incerta* and *Chlamydomonas schloesseri* and *Dunaliella salina*, *Gonium pectorale*, *Tetrabaena socialis*, and *Volvox carteri*), Sphaeropleales (*Monoraphidium neglectum*, *Raphidocelis subcapitata, Scenedesmus obliquus*, *Tetradesmus deserticola*, and *Tetradesmus obliquus*), Chlorellales (*Auxenochlorella protothecoides*, *Chlorella sorokiniana, Chlorella variabilis* and *Helicosporidium* sp.), Trebouxiales (*Coccomyxa subellipsoidea* and *Trebouxia* sp.), Chloropicales (*Chloropicon primus*), Chlorodendrales (*Tetraselmis striata*) and Mammiellales (*Bathycoccus prasinos, Micromonas commoda*, *Ostreococcus lucimarinus* and *Ostreococcus tauri*). The candidate proteins were identified as SWEETs following confirmation of the gene ID/genome location in the JGI PhycoCosm database (Nordberg et al., 2013), the identification of at least one copy of the MtN3/slv Pfam domain (PF03083) and/or the presence of a PQ-loop repeat Pfam domain (PF04193; found predominantly in SemiSWEET sequences) using a Pfam annotation search (Mistry et al., 2020), and the presence of at least three TM helices (forming one THB) using TOPCONS membrane topology prediction software (Tsirigos et al., 2015). The protein sequences were also confirmed as being members of the SWEET family by performing multiple sequence alignments using ClustalW against the well-characterised SWEET representatives from *A. thaliana* described above. Genome details and individual ID numbers or accession numbers for the protein sequences identified and used for subsequent analysis are provided in Supplementary Table 1. For many of these genomes, the transcript details have been supported by transcriptome data from a large number of RNA-Seq datasets; however, these sequences should be considered as representative Chlorophyta SWEETs and it is likely that the exact number of predicted SWEET proteins from some of these genomes may change during subsequent genome reanalysis due to incorrect annotation or genome assembly errors that can lead to genes and protein sequences being missed or incorrectly assigned.

### Identification of outlier and comparative SWEETs

For the phylogenetic analysis of Chlorophyta SWEETs, several fungal and plant SWEETs were used as points of comparison. Unicellular fungal SWEETs allow for comparison of SWEETs from single celled organisms that are not derived from unicellular algae. The chosen fungal SWEETs were six SWEET proteins from *Allomyces macrogynus* (Jia et al., 2017), and nine SWEET proteins from *Neocallimastix californiae* (Podolsky et al., 2021). Lower plant SWEETs from taxa that bridge the evolutionary gap between Chlorophyta and Streptophyta were selected as outliers: eight SWEET proteins from *Amborella trichopoda,* seven from *Selaginella moellendorffii,* two from *Marchantia polymorpha* and one each from *Physcomitrella patens* and *Sphagnum fallax.*

### Phylogenetic analysis

Phylogenetic analysis was carried out using the CIPRES Science Gateway Toolkit (Miller et al., 2010). First, a multiple sequence alignment of the Chlorophyta and fungal SWEETs and the Streptophyta outliers was generated using ClustalW (using default settings). Random Accelerated Maximum Likelihood (RAxML) Black Box was then used to generate a phylogenetic tree (Kozlov et al., 2019), using recommended parameters (Stamatakis, 2014), which included a condition to let RAxML halt bootstrapping following the end of a set run time (30 min; Pattengale et al., 2009), which generated 650 bootstrap replications. Bootstrap percentage values for each node are shown on the tree. Other parameters included use of the BLOSUM62 substitution matrix, the Gonnet 250 matrix for pairwise alignment and no empirical base frequencies. RAxML Black Box automatically generated the best-likelihood tree, based on maximum likelihood, which was evaluated and optimised under GAMMA (0.1 log likelihood). Use of the BLOSUM62 substitution matrix was evaluated using ModelTest-NG (Darriba et al., 2020) and was found to have a high relative scoring log-likelihood value, which was equivalent or better than other substitution models that were also evaluated (lnL scores of −47784.8 for BLOSUM62 compared to −47690.9 for VT, −47737.5 for PMB, −47813.9 for WAG and −47936.9 for LG, according to the Akaike information criterion). Furthermore, it was found that use of other substitution models did not alter the tree structure. For example, comparison of the BLOSUM62 and the LG substitution matrix model found that the structure of both sets of trees was virtually identical, with minor differences in bootstrap values at the nodes (Supplementary Figure 1). In addition, the BLOSUM62 model was preferred to allow consistency with its use in pairwise-similarity CLANS analysis (see below). FigTree^1^ was used to generate tree images.

The Chlorophyta-fungal clade structure was determined by the maximum likelihood tree bootstrap values, then distinguished further by use of pairwise similarity Cluster Analysis of Sequences (CLANS) using CLANS Jar from the MPI Bioinformatics Toolkit (Frickey and Lupas, 2004; Gabler et al., 2020), which allowed determination of specific clusters within the Chlorophyta-fungal clade. The “attractive force” between SWEET proteins using E-values from the BLAST high-scoring pairs was calculated, with the lower the E-value demonstrating the greater the attractive force. Clustering of sequences was calculated by normalising “attractive force” to *p*-values between 0 and 1 (with 1 being a strong cluster and 0 being a weak cluster). The *p*-value was set to 1 × 10^−5^ and the analysis was run for 50,000 iterations, as previously found to be appropriate for phylogenetic validation (Ibuot et al., 2020). CLANS displays the attractive force visually in the Fruchterman-Reingold graph, whereby the stronger attractive forces (the higher normalised p-value, or lower E-value) between specific SWEET proteins are denoted by a darker connective line in the two-dimensional network representation.

### Subcellular localisation prediction

Subcellular localisation prediction was carried out on all Chlorophyta SWEETs using DeepLoc, MULocDeep and PProwler software (Bodén and Hawkins, 2005; Almagro Armenteros et al., 2017; Jiang et al., 2021). These tools were chosen in part due to their ability to accurately be able to confirm the localisation of the *A. thaliana* SWEETs for which membrane localisation has been experimentally determined. A consensus prediction of Chlorophyta SWEET localisation based on the outputs of the three approaches was determined. Predictions with a confidence score > 30% were recorded unless there was no high confidence prediction, in which case the prediction with the highest score was recorded.

### Protein structure modelling and analysis

Protein structure predictions of representative SWEET proteins from Chlorophyta Clusters 1 to 5 were simulated using Phyre-2 homology modelling software (Kelley et al., 2015). In each case Phyre-2 gave the prediction with the highest likelihood (100% confidence in all models; combination of the highest sequence identity and sequence coverage), which was based on the previously solved crystal structure of AtSWEET13 with a substrate analogue, 2′-deoxycytidine 5′-monophosphate (DCM) ligand (PDB id: 5XPD; DOI: 10.2210/pdb5XPD/pdb; Han et al., 2017). The defined protein structure of AtSWEET13 was visualised (Figure 1B) using SWISS-MODEL protein homology software (Waterhouse et al., 2018).

Characterisation of the TM helices and their location in the structure was carried out using TOPCONS (Tsirigos et al., 2015). Conservation across protein sequences was first visualised by Constraint Based Alignment Tool (COBALT; Papadopoulos and Agarwala, 2007) using a frequency-based difference between sequences (BLAST E-value 0.003, Gap penalties −11, −1, End gap penalties −5, −1). Conserved amino acids were visualised using Easy Sequencing in PostScript (ESPript; Robert and Gouet, 2014), with standard default parameters. To demonstrate substrate binding for candidate Chlorophyta SWEET proteins, the Phyre-2 models were run through UCSF Chimera’s in-built docking tool, AutoDock Vina (Trott and Olson, 2010; Gaillard, 2018; Nguyen et al., 2020). Using a PDB file generated from the 5XPD AtSWEET13 template, the substrate-binding pocket was estimated. AutoDock Vina predicted the ligand-binding site based on the orientation positioning of the ligand following simulated binding. Models of DCM ligands bound in representative Cluster 1 to 5 SWEET proteins were determined to be the most accurate if they possessed the most negative ligand binding score, and root-mean-square-deviation values closest to zero.

## Results and discussion

### Phylogenetic relationship of SWEETs in green algae

To investigate the phylogenetic and structural characteristics of SWEET proteins from Chlorophyta, protein sequences were obtained from several databases using selected SWEET protein query sequences and filtered to confirm their identification as SWEETs, including the presence of at least one and mostly two copies of the MtN3/slv Pfam domain (PF03083) in all proteins. This screen identified 70 SWEET proteins of Chlorophyta origin from 28 representative algal strains (Supplementary Table 1). Forty-nine of the proteins were from 15 different Chlorophyceae (order Chlamydomonadales and Sphaeropleales) strains, including five *Chlamydomonas* species, two *Tetradesmus* species and algae such as *V. carteri* and *D. salina*. Thirteen proteins were identified from seven Trebouxiophyceae (order Chlorellales and Trebouxiales) strains, including three *Chlorella* strains, four proteins were from four different Mamiellophyceae (order Mamiellales) strains and four proteins were from Chloropicophyceae (order Chloropicales) and Chlorodendrophyceae (order Chlorodendrales) strains (Table 1). Two of the proteins (ApSWEET2 from *A. protothecoides* and MnSWEET2 from *M. neglectum*) contained just one THB suggesting that these could be SemiSWEET-like proteins although further experimental validation of the sequences will be needed to confirm this is correct or whether they are partial length sequences. The rest of the sequences show characteristics of full-length SWEET proteins. Eleven of the Chlorophyta genomes appeared to possess only one SWEET sequence while the other algal genomes examined encoded between two and six SWEET proteins. This contrasts with the large numbers (usually >20) of SWEET protein isoforms typically present in a higher plant genome. Vascular plants have more complex genomes, caused by occurrence of whole-genome duplication events, leading to the expansion and diversification of SWEET families in higher plants. This allows plants to meet the requirement for more complex sugar transport processes within different cell and tissue types of these large multicellular organisms (Li et al., 2018). In contrast, unicellular algae typically have smaller genome sizes and do not require multiple transporters to mediate sugar translocation across many cell layers, and hence fewer SWEET homologues are observed per species.

**Table 1:** Numbers and distribution of algae (Chlorophyta) and selected fungi (Blastocladiomycota and Chytridiomycota) SWEET proteins.

Order	Species	C1	C2	C3	C4	C5	UC	Total
**Chlorophyta**								
Chlamydomonadales	*Chlamydomonas debaryana*	2	2	1	0	0	0	5
	*Chlamydomonas eustigma*	1	1	1	0	0	0	3
	*Chlamydomonas incerta*	2	1	2	0	0	0	5
	*Chlamydomonas reinhardtii*	2	1	1	0	0	0	4
	*Chlamydomonas schloesseri*	2	2	2	0	0	0	6
	*Dunaliella salina*	0	1	1	0	0	0	2
	*Gonium pectorale*	2	1	1	0	0	0	4
	*Haematococcus lacustris*	1	0	0	0	0	0	1
	*Tetrabaena socialis*	2	0	0	0	0	0	2
	*Volvox carteri*	2	1	1	0	0	0	4
Chlorellales	*Auxenochlorella protothecoides*	0	0	0	0	0	3	3
	*Chlorella sorokiniana* (1228)	0	0	0	0	0	4	4
	*Chlorella sorokiniana* (UTEX 1602)	0	0	0	0	0	1	1
	*Chlorella variabilis*	0	0	0	0	0	1	1
	*Helicosporidium* sp.	0	0	0	0	0	1	1
Chlorodendrales	*Tetraselmis striata*	0	0	0	0	0	3	3
Chloropicales	*Chloropicon primus*	0	0	0	0	0	1	1
Mamiellales	*Bathycoccus prasinos*	0	0	0	0	0	1	1
	*Micromonas commoda*	0	0	0	0	0	1	1
	*Ostreococcus lucimarinus*	0	0	0	0	0	1	1
	*Ostreococcus tauri*	0	0	0	0	0	1	1
Sphaeropleales	*Monoraphidium neglectum*	0	0	0	0	0	2	2
	*Raphidocelis subcapitata*	0	0	1	0	0	1	2
	*Scenedesmus obliquus*	0	0	1	0	0	0	1
	*Tetradesmus deserticola*	0	0	1	0	0	2	3
	*Tetradesmus obliquus*	0	0	1	0	0	4	5
Trebouxiales	*Trebouxia* sp.	0	0	0	0	0	2	2
	*Coccomyxa subellipsoidea*	0	0	0	0	0	1	1
**Blastocladiomycota**								
Blastocladiales	*Allomyces macrogynus*	0	0	0	0	4	2	6
**Chytridiomycota**								
Neocallimastigales	*Neocallimastix californiae*	0	0	0	7	0	2	9

Distribution of SWEET proteins from each analysed genome into five clusters (C1–C5) or classified as “unclustered” (UC), as determined by phylogenetic analysis and supported by CLANS analysis.

**Figure 2:**
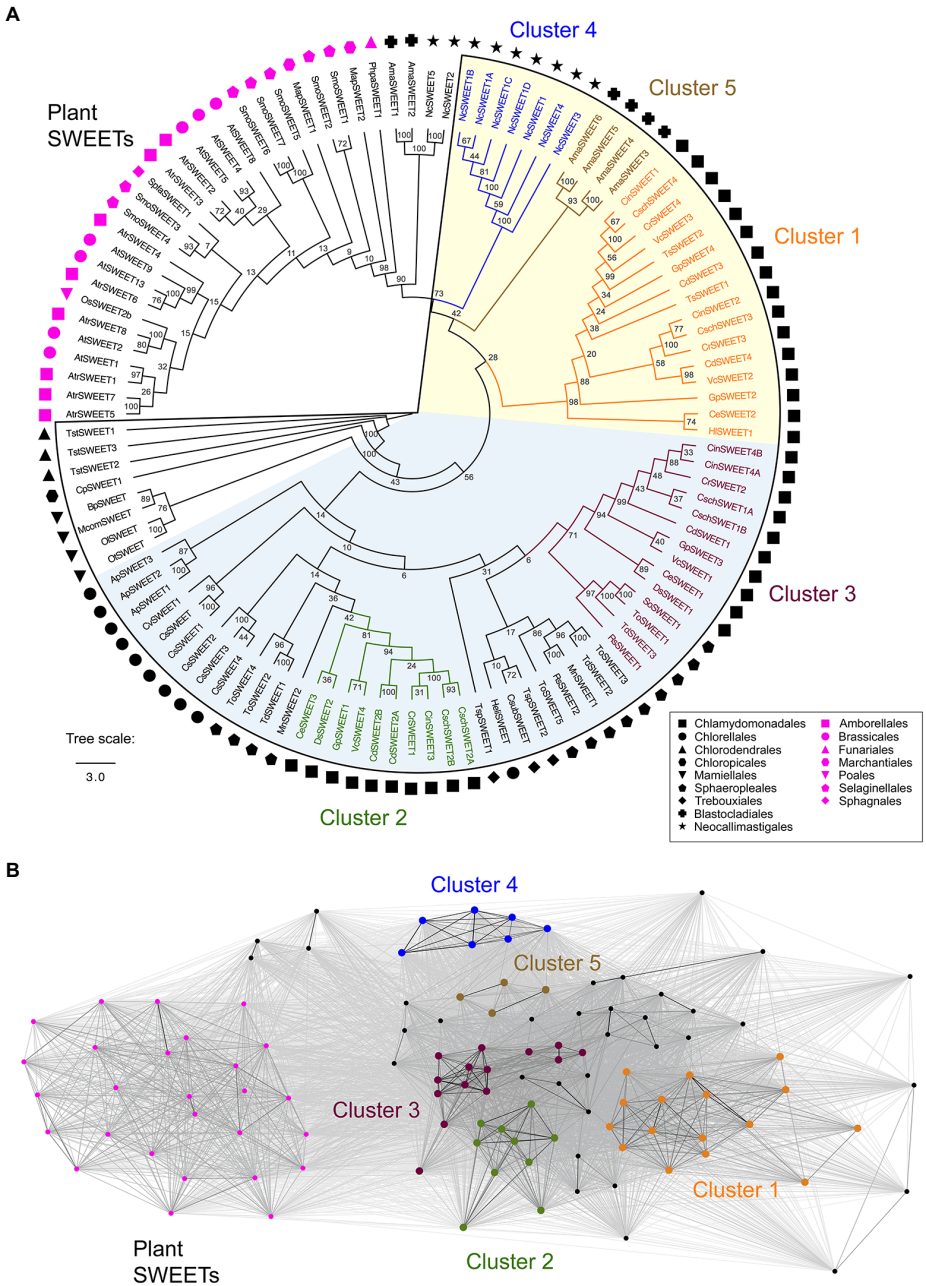
**(A)** Phylogenetic tree of selected Chlorophyta SWEET protein sequences and outliers from representative plants and fungi. The outlined region denotes the Chlorophyta-fungal clade although four fungal-derived SWEETs (NcSWEET2, NcSWEET5, AmaSWEET1, AmaSWEET2) sit outside the clade. Symbols indicate the taxonomic orders of the species where the protein sequence is derived. A consensus maximum likelihood tree following bootstrap replications is shown. The Chlorophyta-fungal clade can be further divided into two sub-clades, determined on the basis of bootstrap percentage values shown at each node, and denoted by the shaded sections of the tree. Algae-fungi SWEETs that form distinct clusters, determined on the basis of CLANS analysis, are highlighted and labelled. The branch length scale bar indicates the evolutionary distance of amino acid substitutions per site. **(B)** A two-dimensional cluster analysis of the SWEET sequences used for the phylogenetic tree was performed using CLANS. Each symbol represents one of the SWEET proteins and is coloured on the basis of the phylogenetic groups shown in Panel A. Grey lines represent protein connections with reciprocal BLAST hits at *p* < 10^−5^. A version of Panel B showing the names of the un-clustered proteins is shown in Supplementary Figure 2. The full list of SWEET sequences and their identification numbers is shown in Supplementary Table 1.

Before constructing a phylogenetic tree, outlier sequences were selected to aid understanding of the evolutionary relationships between the Chlorophyta SWEET proteins and the Streptophyta SWEETs. Previous analysis of SWEET proteins from plant lineages has identified SWEETs from several Bryophyte, Lycophyte, and basal Streptophyta that link to the Chlorophyta SWEETs, but are excluded from the four higher plant SWEET clades (Jia et al., 2017; Li et al., 2018). Representatives of these “linking” SWEETs were therefore selected as additional outliers: eight SWEET protein sequences from the plant *A. trichopoda* that is the most basal lineage of angiosperms, seven from the Lycophyte *S. moellendorffii*, two from the non-vascular plant *M. polymorpha* and one each from the Bryophytes *P. patens* and *S. fallax.* One rice and seven *A. thaliana* SWEET sequences were included in the tree, as Streptophyta representatives, and final outliers to the Chlorophyta-fungal cluster. For comparison, SWEETs from two fungi species were additionally used in order to understand the conservation of SWEETs across these two classes of distinct eukaryotes and help analyse the function of these transporters within less complex organisms. Nine SWEET sequences were taken from *N. californiae* that is ancestral to *Saccharomyces cerevisiae*, and six from the model fungus *A. macrogynus* (Jia et al., 2017; Podolsky et al., 2021). In total 112 SWEETs from selected higher and lower land plants, green algae and anaerobic fungi were used for the phylogenetic tree construction (Figure 2).

A clear phylogenetic distinction was observed between the Chlorophyta SWEETs and the Streptophyta SWEETs including those from Bryophytes and Lycophytes (Figure 2A). In contrast, most of the fungi SWEETs examined, except for two *N. californiae* and two *A. macrogynus* SWEETs (NcSWEET2 and 5; AmaSWEET1 and 2), clustered with all the Chlorophyta SWEETs, forming an Chlorophyta-fungi clade (Figure 2A). The conservation of fungi-derived SWEETs within this clade is consistent with previous observations (Jia et al., 2017). Bootstrap values indicate that the Chlorophyta-fungal clade can be further divided into two sub-clades (Figure 2A; Supplementary Figure 1). Phenetic sequence clustering analysis by CLANS was used to provide further distinction of the tree structure and to support the classification of clusters of specific proteins within the clade. CLANS determined the “attractive force” between SWEET protein sequences by performing an all-against-all pairwise similarity search (Frickey and Lupas, 2004). CLANS confirmed the separation of the four outlier fungal SWEETs, which were distinct from plant SWEETs and the rest of the Chlorophyta-fungal clade (Figure 2B). CLANS also supported the grouping of phylogenetically similar SWEET proteins into five clusters within the clade named Cluster 1 to 5 (Figure 2). Furthermore, this CLANS method was used to identify individual protein sequences that did not show strong clustering due to a low attractive force value with members of a cluster, and therefore were not included in one of the five clusters (Supplementary Figure 2). For example, sequence MnSWEET2 was very distant from the 10 Cluster 2 proteins in the CLANS network and so was not included in Cluster 2.

Cluster 1 and 2 are solely composed of proteins derived from the Chlamydomonadales order, whereas Cluster 3 includes both Chlamydomonadales and Sphaeropleales representatives. Clusters 4 and 5 specifically distinguish the fungi within the clade, with *N. californiae* SWEETs in Cluster 4 and *A. macrogynus* SWEETs in Cluster 5 (Figure 2). All the remaining Sphaeropleales SWEETs as well as all of the SWEET sequences derived from the Trebouxiophyceae, Chloropicophyceae, Chlorodendrophyceae and Mamiellophyceae classes of algae could not be grouped within defined clusters supported by CLANS analysis, and so were defined as “un-clustered.” While this phylogenetic analysis indicates that many of the SWEET sequences have clustered by higher order taxonomy, there is also a clear separation of several SWEETs from the same species into different clusters. For example, SWEETs from *C. reinhardtii*, *G. pectorale* and *V. carteri* are present in Clusters 1, 2, and 3, indicating potential functional and structural differences between the proteins, as is the case for the higher plants SWEETs. However, since there is currently no experimental characterisation of Chlorophyta SWEETs, confirmation of functional and structural differences must be initially derived from bioinformatics analyses.

### Subcellular localisation of green algae SWEETs

Subcellular localisation prediction by a combination of algorithms was utilised to further characterise the Chlorophyta SWEETs and discern if there was commonality between proteins in the same cluster. DeepLoc and MULocDeep algorithms were used to indicate localisation to specific plasma membrane or organelle membranes by the presence of a signal peptide (Almagro Armenteros et al., 2017; Jiang et al., 2021). In contrast, PProwler, known for its high accuracy and sensitivity for predicting localisation in green algae (Bodén and Hawkins, 2005), complemented the predictions with indications of localisation to secretory pathway membranes (including plasma membrane, tonoplast, endoplasmic reticulum or Golgi), the mitochondrion, chloroplast or “other” (including nucleus and cytosol). To ensure an accurate estimation of localisation, a prediction was made based on the consensus of the three algorithms (Table 2). In the cases where there was significant likelihood (30%–50% score) of localisation to another membrane, or in cases where one algorithm clearly predicted localisation to another membrane, a secondary or tertiary prediction was also recorded.

**Table 2:** Subcellular localisation predictions of Cluster 1 to 3 Chlorophyta SWEETs.

Cluster	SWEET protein	DeepLoc	MuLocDeep	PProwler	Consensus
1	CdSWEET3	PM (65)	PM (61)	SP (61)	PM
1	CdSWEET4	PM (81)	PM (66)	SP (82)	PM
1	CeSWEET2	PM (79)	PM (56)	SP (74)	PM
1	CinSWEET1	PM (70)	PM (46)	SP (38)/Mit (48)	PM/Mit
1	CinSWEET2	PM (64)	PM (47)	SP (55)/Mit (34)	PM/Mit
1	CrSWEET3	PM (81)	PM (47)	SP (77)	PM
1	CrSWEET4	PM (81)	PM (51)	SP (92)	PM
1	CschSWEET3	PM (65)	PM (53)	SP (71)	PM
1	CschSWEET4	PM (86)	PM (77)	SP (92)	PM
1	GpSWEET2	PM (81)	PM (54)	SP (94)	PM
1	GpSWEET4	PM (68)	PM (66)	SP (98)	PM
1	HlSWEET	PM (41)/VM (51)	PM (43)	SP (96)	PM/VM
1	TsSWEET1	PM (83)	PM (62)	SP (95)	PM
1	TsSWEET2	PM (66)	PM (29)	SP (64)	PM
1	VcSWEET2	PM (85)	PM (86)	SP (64)	PM
1	VcSWEET3	PM (75)	PM (48)	SP (64)	PM
2	CdSWEET2A	PM (80)	PM (72)	SP (78)	PM
2	CdSWEET2B	PM (80)	PM (60)/VM (31)	SP (79)	PM/VM
2	CeSWEET3	PM (42)/VM (55)	PM (56)	SP (88)	PM/VM
2	CinSWEET3	PM (46)	PM (43)	SP (64)	PM
2	CrSWEET1	PM (87)	PM (53)	SP (59)/O (35)	PM/O
2	CschSWEET2A	PM (90)	PM (51)	SP (59)/O (33)	PM/O
2	CschSWEET2B	PM (85)	PM (61)	SP (60)/O (33)	PM/O
2	DsSWEET2	PM (83)	PM (48)	SP (65)	PM
2	GpSWEET1	PM (87)	PM (64)	SP (70)	PM
2	VcSWEET4	PM (92)	PM (79)/VM (32)	SP (81)	PM/VM
3	CdSWEET1	PM (35)/VM (59)	PM (58)/VM (36)	O (80)	PM/VM/O
3	CeSWEET1	PM (65)/VM (34)	PM (80)	SP (69)	PM/VM
3	CinSWEET4A	PM (51)/VM (43)	VM (35)	O (79)	PM/VM/O
3	CinSWEET4B	PM (49)/VM (38)	VM (25)	O (79)	PM/VM/O
3	CrSWEET2	PM (49)/VM (46)	PM (47)	O (76)	PM/VM/O
3	CschSWEET1A	PM (61)/VM (34)	VM (41)	O (83)	PM/VM/O
3	CschSWEET1B	PM (48)/VM (45)	PM (31)/VM (37)	O (83)	PM/VM/O
3	DsSWEET1	PM (55)	PM (71)	SP (64)	PM
3	GpSWEET3	VM (37)	PM (40)	SP (64)	PM/VM
3	RsSWEET1	PM (54)/VM (44)	PM (60)	SP (79)	PM/VM
3	SoSWEET1	PM (89)	PM (74)	SP (64)	PM
3	TdSWEET3	PM (50)/VM (49)	PM (66)	SP (64)	PM/VM
3	ToSWEET1	PM (89)	PM (77)	SP (64)	PM
3	VcSWEET1	PM (46)/VM (45)	PM (39)/VM (47)	O (74)	PM/VM/O

The DeepLoc and MULocDeep outcomes include: endoplasmic reticulum (ER), Golgi (G), plasma membrane (PM) or vacuolar membrane (VM), and the PProwler outcomes include: mitochondria (Mit), other (O) or secretory pathway (SP). Only the highest confidence score (%) value predictions with a score > 30% are shown except when there were only low score predictions.

While most of the algal SWEET sequences have plasma membrane as the only significant prediction or the primary prediction, there was variation between SWEET homologues from the same species and between SWEETs from different clusters, potentially indicative of differences in protein function. Predicted localisation to the plasma membrane was conserved across all Cluster 1 SWEETs, except for CinSWEET1, which is moderately likely to localise to the mitochondrial membrane according to PProwler, or HlSWEET, which may localise to the vacuolar membrane (Table 2). Although MULocDeep and DeepLoc algorithms shared consensus over plasma membrane localisation predictions for eight out of 10 Cluster 2 SWEETs, PProwler indicated a significant likelihood of localisation to “other” membranes (>30% confidence score) in three Cluster 2 SWEETs (CrSWEET1, CschSWEET2A, and CschSWEET2B; Table 2). As such, consensus predictions indicate that half of Cluster 2 SWEETs are likely to localise to the plasma membrane, while the others have a low likelihood of localisation to the vacuole or “other” membranes (Table 2). Predictions for Cluster 3 SWEETs were less confident, with different algorithms giving secondary or tertiary predictions to the Golgi, vacuole or “other” membranes. Notably, in contrast to Clusters 1 and 2, algorithms offered consensus over vacuole/lysosomal membrane localisation for 5 out of 14 Cluster 3 SWEETs (VcSWEET1, CinSWEET4A, CinSWEET4B, CschSWEET1A, and CschSWEET1B) and confidently predicted plasma membrane localisation in only two of the Cluster 3 SWEETs (ToSWEET1 and SoSWEET1). This suggests that some SWEETs may be responsible for sugar transport across the membranes of a vacuole/lysosome-related organelle rather than the cell membrane. There is limited knowledge of vacuolar transport processes in green algae particularly relating to sugar storage or release. Many chlorophytes possess both a contractile vacuole, with potential roles in osmoregulation and protein degradation (Becker, 2007), and lysosome-like acidocalcisomes that are involved in metal storage and polyphosphate regulation (Goodenough et al., 2019). Although there is some indication of metabolite transport pathways within these organelles, there is currently no clear evidence for the need of a sugar transporter.

DeepLoc has a high sensitivity for plasma membrane localisation but low sensitivity towards vacuole membrane predictions (Almagro Armenteros et al., 2017; Savojardo et al., 2018). To validate the accuracy of these predictions, notably for Cluster 3 proteins where predictions were less certain, DeepLoc and MULocDeep predictions were performed on *A. thaliana* SWEETs (AtSWEET1–17) whose precise intracellular localisation has been experimentally validated (Table 3). In higher plants, Clade I to III SWEETs are broadly characterised by their plasma membrane localisation, whereas Clade IV SWEETs are distinguished by vacuolar localisation (Chen et al., 2010; Eom et al., 2015). In nearly all cases the DeepLoc and MULocDeep predictions of the Clade I – III (AtSWEET1–15) and Clade IV (AtSWEET16–17) SWEETs aligned with the experimentally validated localisation, although for vacuolar localised AtSWEET2 (Chen et al., 2015), the predictions were less confident: a plasma membrane score of 49% (both DeepLoc and MULocDeep) and a vacuole membrane score of 46% (DeepLoc) or 24% (MULocDeep). This indicates that predicting localisation for some vacuolar membrane SWEET proteins is challenging and supports the idea that some of the algal Cluster 3 SWEETs may indeed localise to a vacuole membrane, but of course experimental analysis is needed to fully validate these predictions.

**Table 3:** Subcellular localisation predictions and known location comparisons of Clade I to IV *Arabidopsis thaliana* SWEETs.

Clade	SWEET protein	DeepLoc	MuLocDeep	Known localisation
I	AtSWEET1	PM (72)	PM (88)	PM
I	AtSWEET2	PM (49)/VM (46)	PM (49)	VM
I	AtSWEET3	PM (83)	PM (74)	PM
II	AtSWEET4	PM (94)	PM (91)	PM
II	AtSWEET5	PM (87)	PM (90)	PM
II	AtSWEET6	PM (86)	PM (86)	PM
II	AtSWEET7	PM (95)	PM (88)	PM
II	AtSWEET8	PM (84)	PM (86)	PM
III	AtSWEET9	PM (91)	PM (65)	PM
III	AtSWEET10	PM (88)	PM (71)	PM
III	AtSWEET11	PM (96)	PM (98)	PM
III	AtSWEET12	PM (96)	PM (98)	PM
III	AtSWEET13	PM (92)	PM (97)	PM
III	AtSWEET14	PM (95)	PM (87)	PM
III	AtSWEET15	PM (97)	PM (93)	PM
IV	AtSWEET16	VM (63)	VM (91)	VM
IV	AtSWEET17	PM (31)/VM (61)	VM (85)	VM

The DeepLoc and MULocDeep outcomes include: plasma membrane (PM) or vacuolar membrane (VM). Only the highest confidence score value (%) predictions with a score > 30% are shown.

### Amino acid sequence conservation across green algae and fungi SWEET proteins

**Figure 3:**
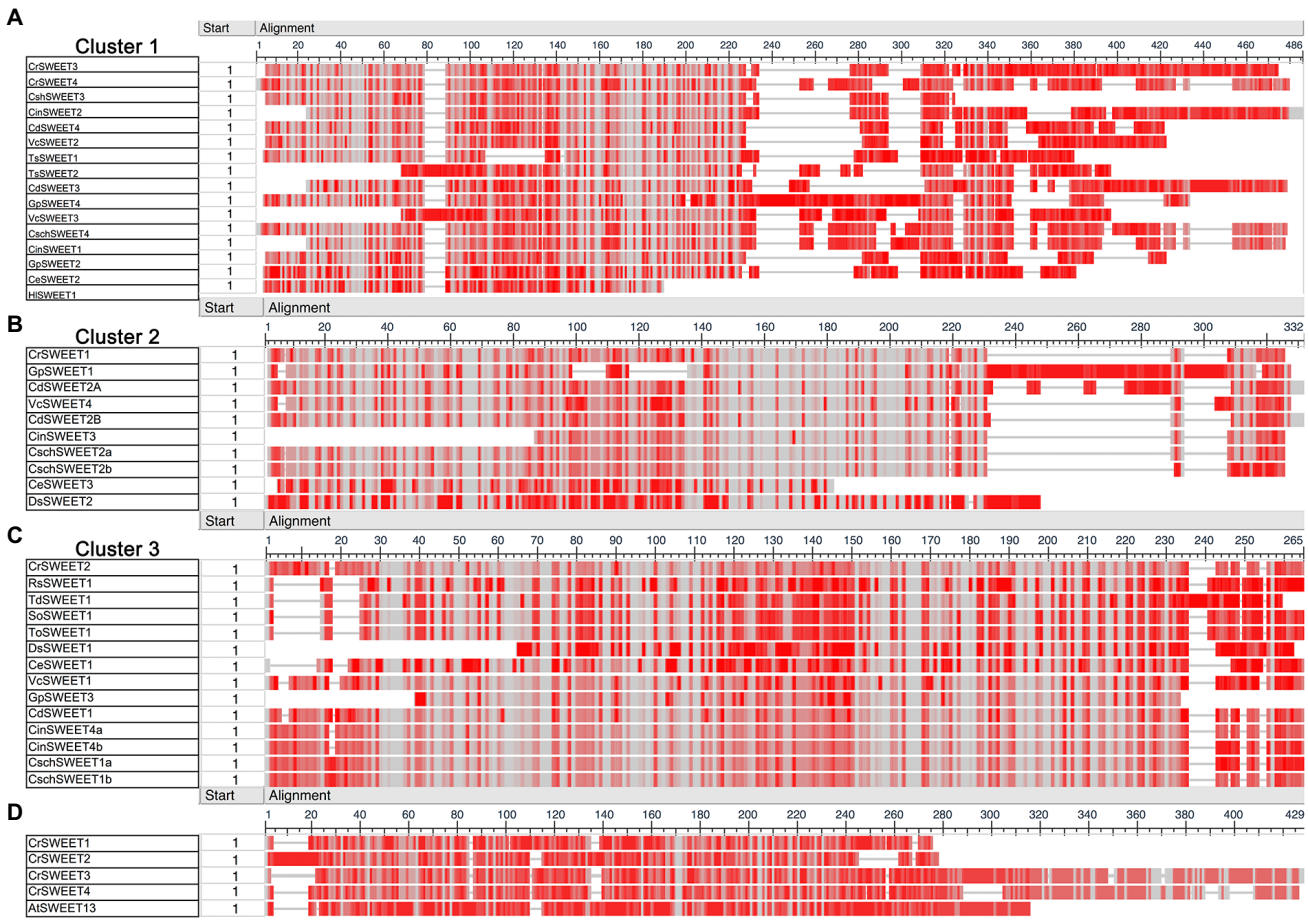
**(A–C)** COBALT multiple sequence alignment of Cluster 1 **(A)**, Cluster 2 **(B),** and Cluster 3 **(C)** algae SWEET protein sequences. **(D)** Comparison of AtSWEET13 with CrSWEET1 to 4. Amino acid conservation was scored based on frequency within each amino acid position (column). Grey shading indicates and identical residue at that position and darker shades of red indicate differences from residues in other rows in the alignment at that position.

Multiple sequence alignments were carried out for the Cluster 1 to 5 sequences to visualise conservation between sequences *via* a “frequency-based difference” approach. Amino acids were scored based on the frequency of their representation in each column with the infrequent (low conservation) amino acids highlighted with darker shading. Comparison of the three algal clusters (Cluster 1, 2, and 3) found that while there are regions of high conservation within each cluster, there are noticeable differences between clusters (Figure 3). Cluster 1 SWEETs, which includes CrSWEET3 and CrSWEET4, all have an extensive, non-conserved C-terminal region (Figure 3A), which was absent from Cluster 2 and 3 proteins (Figures 3B,C). The C-terminal tail regions of SWEETs are predicted to reside on the cytosolic side of the membrane and may provide a docking platform for protein interactions. This allows oligomerisation to form homo- or hetero-dimers, or interactions with other proteins, such as kinases for phosphorylation to regulate sugar transport across the membrane (Eom et al., 2015; Han et al., 2017; Chen et al., 2022). The Cluster 2 proteins (which include CrSWEET1) and Cluster 3 proteins (which include CrSWEET2) show higher amino acid conservation than Cluster 1 proteins (Figures 3A–C), in part due to the lack of a long variable C-terminal tail, but also likely due to several proteins in this cluster all deriving from the *Chlamydomonas* genus and being orthologues of one another. The two fungal clusters (Cluster 4 and 5) showed very high sequence conservation, due to a small sample size within the Cluster and because all sequences were derived from the same species (Supplementary Figure 3).

**Figure 4:**
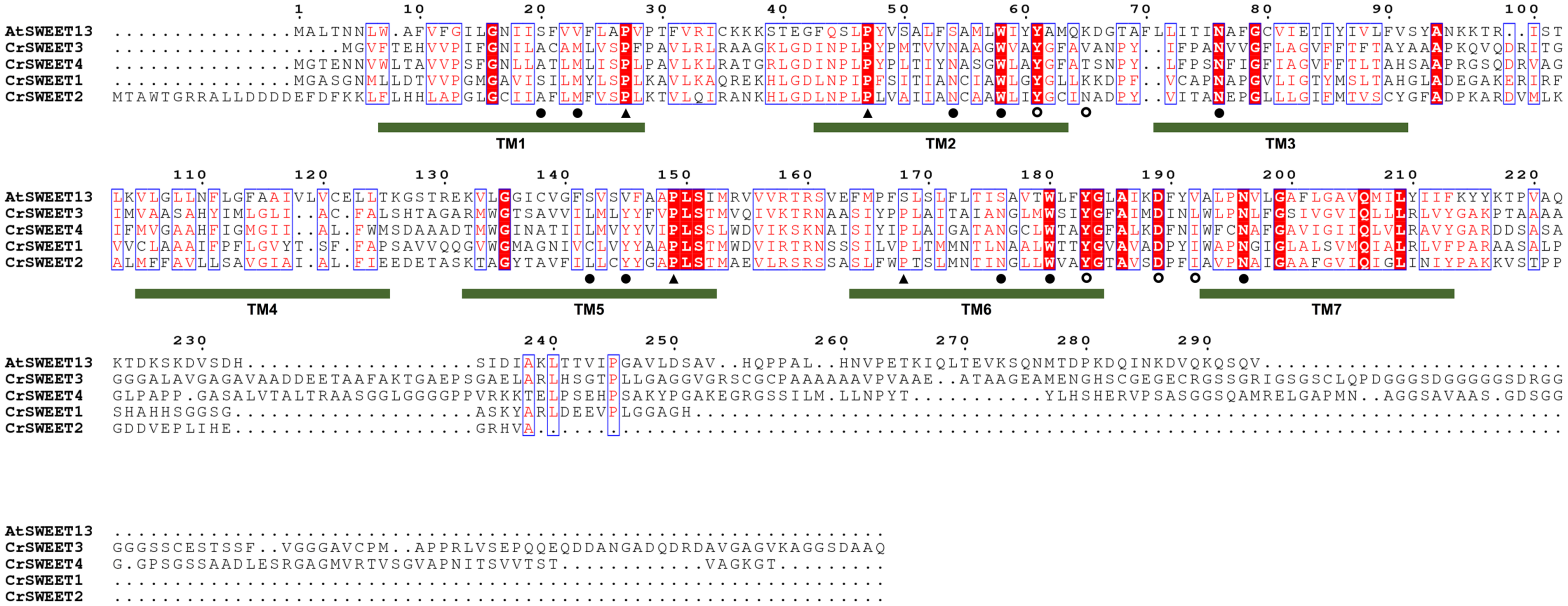
Multiple amino acid sequence alignment of AtSWEET13 compared to CrSWEET1 to 4. Amino acids that are identical or similar are in red shading or red font, respectively. Critical residues in AtSWEET13 that are required for substrate selectivity (filled circles) and transport mechanism, either as extrafacial hinge residues (open circles) or residues required for conformational change (closed triangles) are indicated with a symbol under the sequence. The position of the TM helices for AtSWEET13 is indicated and the numbering is based on the AtSWEET13 sequence.

Comparison of the *C. reinhardtii* SWEETs (CrSWEET1 to 4) with the well-characterised AtSWEET13 protein (Figure 3D) indicates discrete regions of extremely high conservation between the sequences. In particular, there are 21 residues that are identical between the four *C. reinhardtii* SWEETs and AtSWEET13 and other residues that are similar (Figure 4). This may indicate that some or all of these residues are of critical importance to SWEETs, and therefore functional characterisation at an amino acid level is necessary, which requires understanding of the tertiary structure of the Chlorophyta SWEETs.

### Structural simulation of green algae and fungi SWEETs

**Figure 5:**
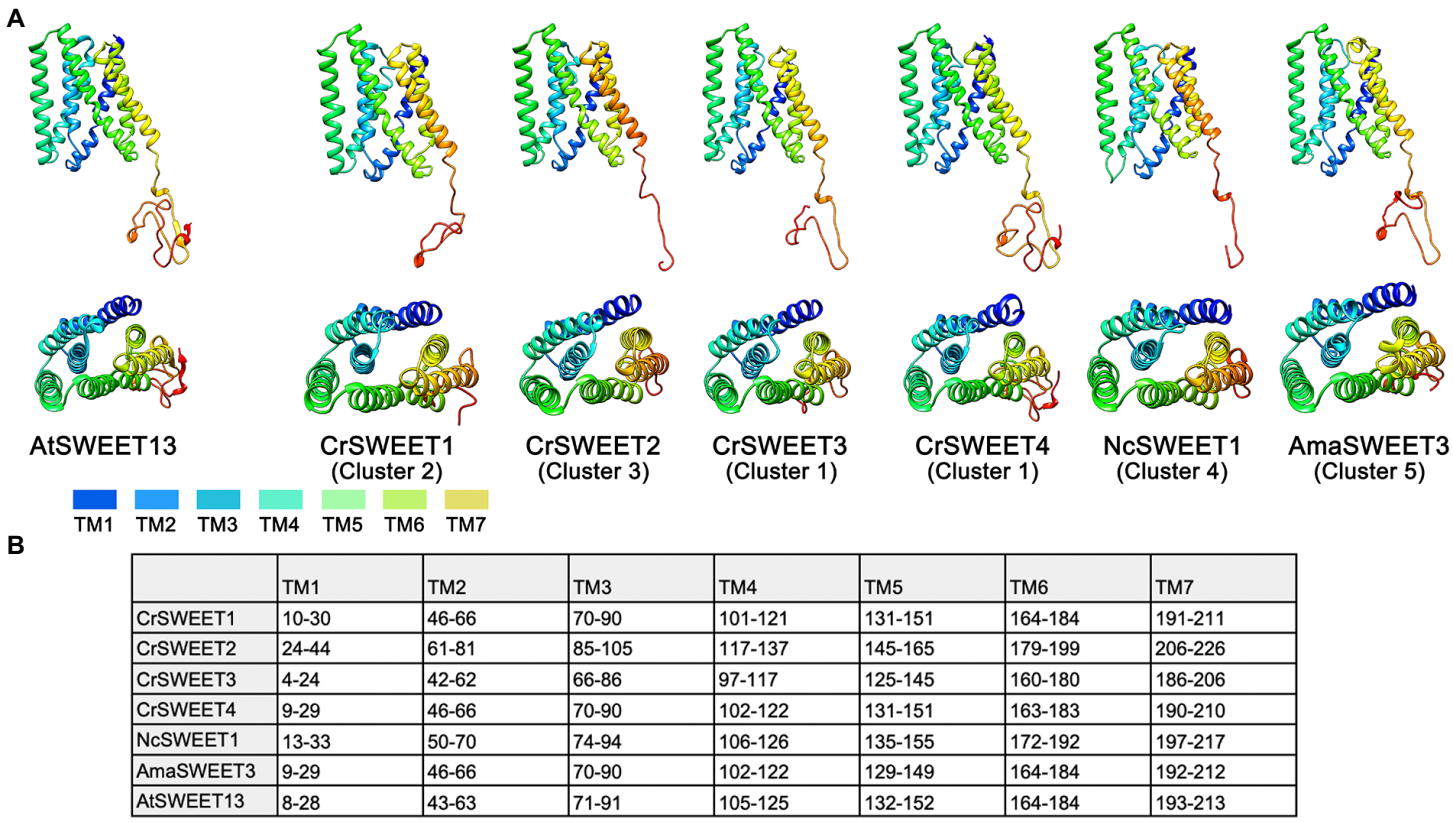
**(A)** Structure models of representative Cluster 1 to 5 algae and fungi SWEETs compared to the known protein structure of AtSWEET13 (PDB ID: 5XPD) that was used as the template for modelling. Ribbon structures of Cluster 1 (CrSWEET3 and CrSWEET4), Cluster 2 (CrSWEET1), Cluster 3 (CrSWEET2), Cluster 4 (NcSWEET1), Cluster 5 (AmaSWEET3) and template (AtSWEET13) SWEET proteins viewed from within the membrane (top image) and a top-down view from the extrafacial side (bottom image). TM helices are denoted by different colours. **(B)** Predicted location determined by TOPCONS for the seven TM helices of each SWEET protein sequence. Numbers refer to amino acid residues.

Understanding the structure or structural differences of algae SWEETs is critical to inform predictions of protein function and mechanism of transport. A template-based homology approach (Phyre-2) was applied to representative SWEET sequences from Clusters 1 to 5, with the assumption that protein structure prediction from solved SWEET-structure templates would give more reliable simulations. Crystal structures have been solved for the angiosperm SWEETs OsSWEET2b and AtSWEET13 (Tao et al., 2015; Han et al., 2017) and bacterial SemiSWEETs, including LbSemiSWEET, EcSemiSWEET, and TySemiSWEET (Wang et al., 2014; Xu et al., 2014; Lee et al., 2015). AtSWEET13 was chosen as the template for homology modelling for two reasons: SemiSWEETs possess only 3 TM helices (only one THB), thus provide both lower confidence alignments and alignments with less sequence coverage; and OsSWEET2b crystal structure was characterised as a homo-3-mer, thus models displayed lower sequence alignment compared to the monomeric structure of AtSWEET13. The modelled structures of the algal CrSWEET3 and 4 (Cluster 1), CrSWEET1 (Cluster 2) and CrSWEET2 (Cluster 3) proteins, the fungal NcSWEET1 (Cluster 4) and AmaSWEET3 (Cluster 5) proteins, alongside the AtSWEET13 template, are shown in Figure 5A. TOPCONS was used to estimate the residue positions of the TM helices of each protein (Figure 5B). The structural simulations show the presence of seven TM domains for each algal and fungal SWEET protein, which is characteristic of the SWEET family (Jia et al., 2017). Furthermore, each of the proteins appeared to emulate the THB structure of AtSWEET13 (Figure 1); namely THB1 and THB2 arranged in a [1-3-2] and [5-7-6] formation, respectively, and linked by a central TM4 helix (Figure 5).

In all cases, Phyre-2 was able to simulate SWEET structure with 100% confidence; however, the SWEETs varied with regard to alignment and homology to the template. CrSWEET1 and CrSWEET2 shared good alignment with the template: 22% identity, 94% coverage and 28% identity, 88% coverage, respectively. In contrast, both CrSWEET3 and CrSWEET4 displayed lower homology and alignment with the template: 21% identity, 61% coverage and 17% identity, 71% coverage, respectively. This could be due to the extensive non-conserved C-terminal tail region, most prominent in CrSWEET3 and 4. However, the fungal AmaSWEET3 (Cluster 5) demonstrated high homology with the template (21% identity, 92% coverage) despite also possessing an extensive non-conserved C-terminal tail (Figure 5A). For homology-based structural predictions, these values represent an accurate alignment. TOPCONS analysis was also able to identify homologous structures to either THB1 or THB2 in each assessed SWEET. Particularly, the THB2 region of CrSWEET1, CrSWEET2, NcSWEET1 and AmaSWEET3 all displayed homology to the single-THB, bacterial SemiSWEET: LbSemiSWEET (PDB: 4QNC). Similarly, the THB2 region of AtSWEET13 displayed homology to the bacterial SemiSWEET TySemiSWEET (PDB: 4rng). Although the process of SWEET evolution is not well characterised, it has been postulated that eukaryotic SWEETs evolved from a duplication and fusion event of individual SemiSWEETs, or a fusion of a bacterial SemiSWEET and archaeal SemiSWEET (Xuan et al., 2013; Hu et al., 2016). Alignment of THB2 in CrSWEET1, CrSWEET2, NcSWEET1 and AmaSWEET3 but not in CrSWEET3 or CrSWEET4, perhaps indicates a divergence of evolution of the latter two proteins.

### Prediction of substrate binding and transport

**Figure 6:**
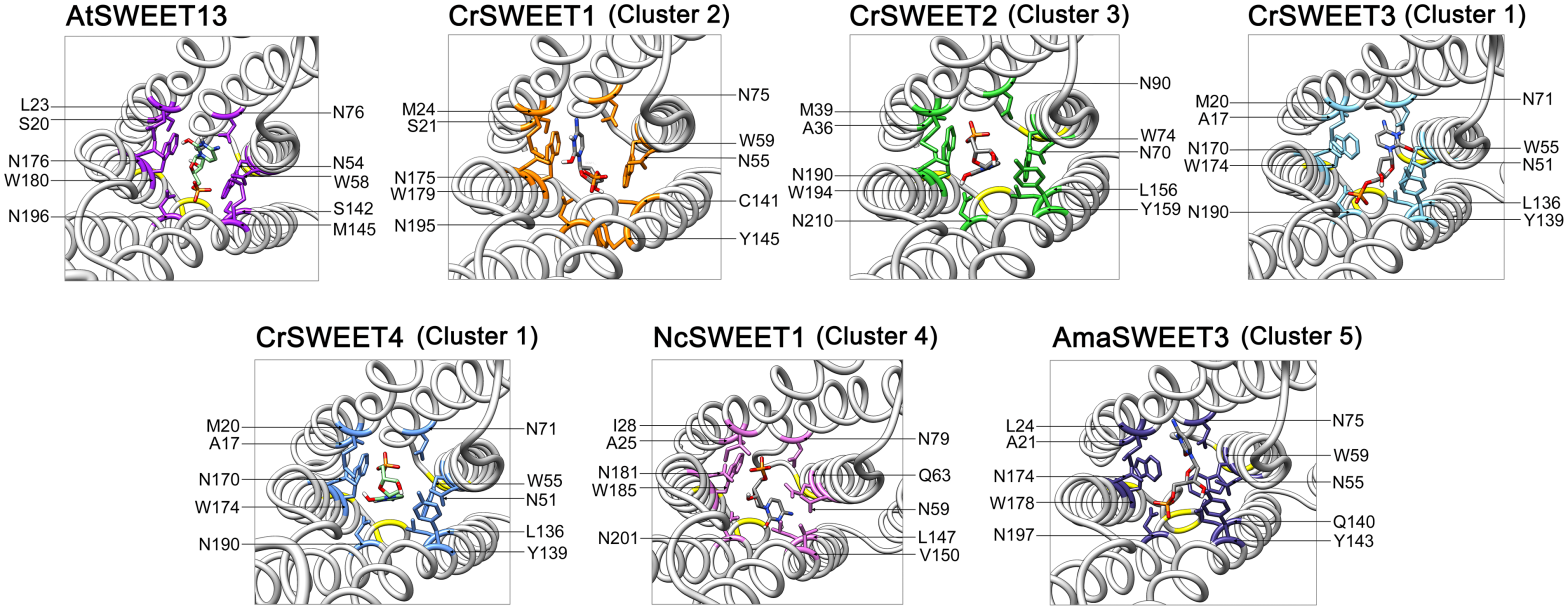
Predicted substrate binding site residues of CrSWEET1 to 4, NcSWEET1 and AmaSWEET3, compared with AtSWEET13. The DCM substrate surrounded by the binding pocket residues is shown. Residues were determined by alignment with the previously characterised binding site residues for AtSWEET13. The structures are viewed from the intrafacial/cytosolic side through the pore channel.

**Figure 7:**
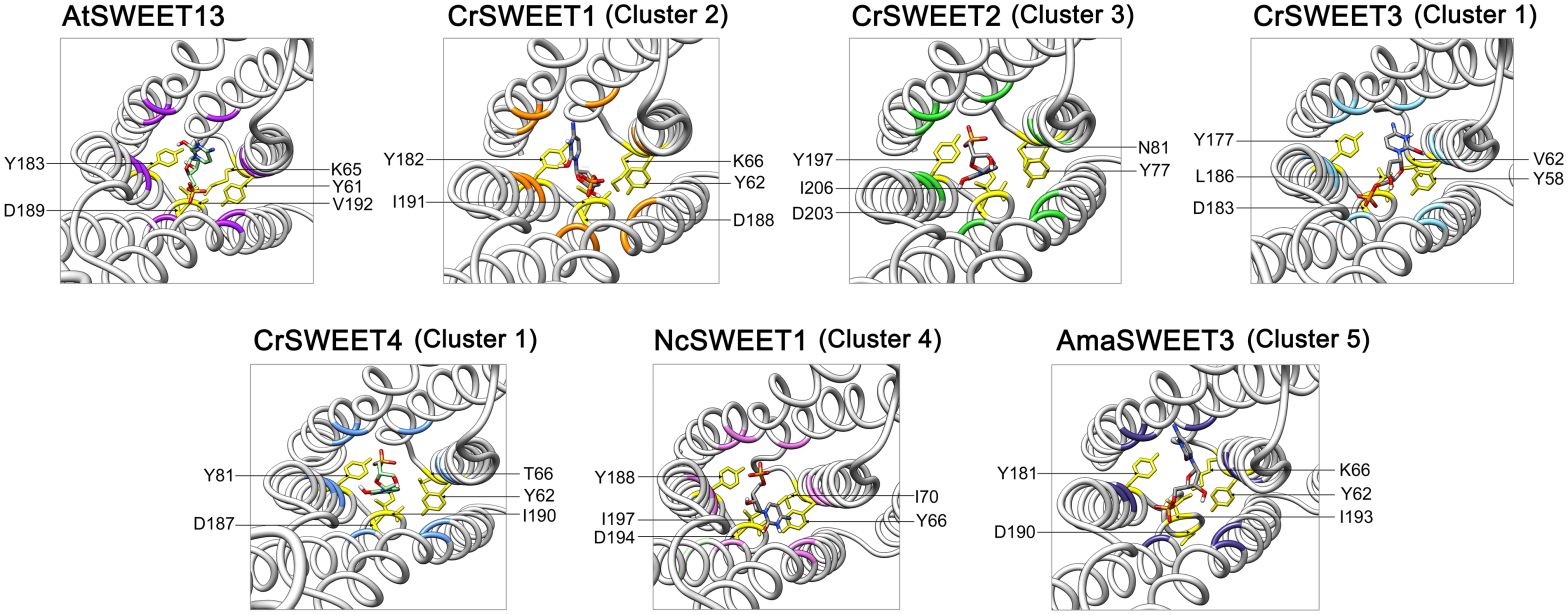
Predicted extrafacial hinge residues of CrSWEET1 to 4, NcSWEET1 and AmaSWEET3, compared with AtSWEET13. The DCM substrate associated with the extrafacial gate residues is shown. Residues were determined by alignment with the previously characterised extrafacial hinge residues for AtSWEET13. The structures are viewed from the lumenal or extracellular side through the pore channel.

Ten hydrophobic and polar residues in a modified version of AtSWEET13, which was used to generate the crystal structure, are crucial for ligand binding and have been shown to comprise the putative substrate binding pocket. These are Ser20, Leu23 (Val23 in wild type AtSWEET13), Asn54 (Ser54 in wild type AtSWEET13), Trp58, Asn76, Ser142, Met145 (Val145 in wild type AtSWEET13), Asn176 (Ser176 in wild type AtSWEET13), Trp180 and Asn196 (Han et al., 2017). Many of these residues are conserved in the *C. reinhardtii* SWEETs within Clusters 1, 2 and 3 (Figure 4), and in Cluster 4 and 5 fungal SWEETs (Supplementary Figure 4). To show how these residues are arranged on the modelled SWEET structures and how they could interact with a substrate, protein-ligand docking simulation was performed. The substrate analogue DCM, which was used to simulate glucose binding within the AtSWEET13 structure (Han et al., 2017), was used for substrate docking simulation in each of the representative Cluster 1 to 5 SWEETs. DCM was predicted to fit within the binding pocket of each SWEET protein although there was variation in the orientation of DCM binding as determined by the highest binding affinity score in each structure simulation (Supplementary Figure 5). By comparing residues in the known binding site and extracellular hinge of AtSWEET13 with the Cluster 1 to 5 SWEETs, it was possible to overlay and identify the critical residues in the representative algal and fungal SWEETs that may be involved in the substrate binding pocket (Figure 6) and extrafacial gate (Figure 7).

With a few notable exceptions, these key residues identified in AtSWEET13 are conserved within the representative algae and fungi SWEET binding pocket region (Figure 6). It has been demonstrated from AtSWEET13 mutation experiments that four binding pocket residues are crucial identifiers for the selection of monosaccharide or disaccharide substrates (Han et al., 2017). Val23, Ser54, Val145, and Ser176 (numbered according to AtSWEET13) determine disaccharide (such as sucrose) selection, while Leu, Asn, Met, and Asn residues in the equivalent positions determine monosaccharide (such as glucose) selection. To simulate glucose transport in the AtSWEET13 crystal structure, four amino acid mutations (V23L, S54N, V145M, S176N) were made to convert the binding pocket from being disaccharide-specific to monosaccharide-specific, resulting in an abolishment of sucrose transport activity but maintenance of glucose transport activity (Han et al., 2017). The Leu, Asn, and Met residues are longer and possess more bulky side chains, which is thought to restrict the size of the binding pocket and prevent larger disaccharide sugar binding (Chen et al., 2010). Leu23 and Met145 do not interact with the ligand directly, but rather stabilise and restrict the cavity size of the binding pocket. In the Cluster 1 to 3 microalgae SWEETs, the equivalent positions are held by a Met residue (e.g.,Met24 in CrSWEET1) and a Tyr residue (e.g., Tyr145 in CrSWEET1; Figure 4), which possess significantly bulkier side chains (Figure 6). This would indicate a further restriction of the size of the binding pocket and the size of the sugar able to be transported. Cluster 4 fungal SWEETs, including NcSWEET1, have a bulkier Ile residue in the Val23 equivalent position (Ile28 in NcSWEET1), but retain the Val residue in the Val145 position (Val150 in NcSWEET1; Figure 6; Supplementary Figure 4). NcSWEET1 has been shown to selectively transport both hexose and pentose sugars, therefore it would appear that the restricted-size binding pocket cavity facilitates the transport of smaller sugar substrates (Podolsky et al., 2021). As such, it is possible that these algae SWEETs as well as the examined fungi SWEETs may only be able to transport small hexose or pentose sugars due to the restricted binding pocket size.

The bulky side chain of Trp residues was thought to stabilise the sugar substrate in the binding pocket (Lee et al., 2015; Han et al., 2017). Comparison of algae SWEET and AtSWEET13 sequences and structures has revealed the conservation of the crucial Trp-Asn pairs (e.g., Trp58-Asn76 and Trp180-Asn196 in AtSWEET13; Trp59-Asn75 and Trp179-Asn195 in CrSWEET1) (Figures 4, 6), indicative of a conserved ligand-binding mechanism. The solved crystal structure of EcSemiSWEET, modelled with monoolein to mimic a sugar substrate, revealed a hydrogen bond between the hydroxyl of the substrate and Asn66 (Asn76 in AtSWEET13). The aromatic group of the adjacent Trp residue, Trp50 (Trp58 in AtSWEET13), stabilised the substrate in the binding pocket (Lee et al., 2015). This process is observed in both DCM and glucose binding by AtSWEET13 and AtSWEET1 (Han et al., 2017; Selvam et al., 2019). The conservation of these critical binding pocket residues across the algae Cluster 1 to 3 SWEETs and the fungal Cluster 5 SWEETs (Supplementary Figure 4), and the conformational similarities between the SWEET simulations, strongly indicates a conserved substrate binding mechanism, despite the potential variation in the orientation of DCM in the binding pockets of each SWEET (Supplementary Figure 5).

The crystal structure of AtSWEET13, used as a template for the Cluster 1 to 5 SWEET modelling, was solved in the inward-facing (IF) state with a monomeric stoichiometry (Han et al., 2017). By aligning conserved residues known to aid in the substrate translocation from the IF to outward-facing (OF) state, we can predict which residues may play a similar role in the sugar transport pathway in the Cluster 1–5 SWEETs. A tetrad of Pro residues (Pro27, Pro47, Pro150, Pro167; numbered according to OsSWEET2b) are highly conserved across eukaryotic SWEETs, SemiSWEETs and Cluster 1 to 5 SWEETs (Figure 4; Lee et al., 2015; Selvam et al., 2019). Pro27 and Pro150 (equivalent to Pro28 and Pro148 in CrSWEET1) induce a 30° kink in TM1 and TM5, respectively (Figure 1C). This kink in the TM helix is an indication of a molecular hinge responsible for the transition from IF to OF state (Lee et al., 2015; Tao et al., 2015). The remaining Pro tetrad residues, Pro47 and Pro167 (equivalent to Pro48 and Pro167 in CrSWEET1) are found to terminate the TM2 and TM6 helices, respectively. These Pro residues are crucial for substrate transport, such that mutation of any of these residues in AtSWEET1 resulted in a complete loss of sugar transport activity (Tao et al., 2015). Adjacent residues are thought to stabilise the substrate while the protein is undergoing conformational change. Glu residues that complete the PQ loop repeat in SemiSWEETs form cross-protomer linkages, while in eukaryote SWEETs, covalent linkages with the TM4 helix replace the role Glu residues (Xu et al., 2014; Lee et al., 2015; Tao et al., 2015).

Clusters of residues in AtSWEET13 that localise to the protein extremities comprise the intra- and extrafacial gates (Han et al., 2017). As the AtSWEET13 structure was solved in the IF state, we can observe residues interacting in the extrafacial gate, including Tyr61, Tyr183, Asp189, Lys65 and Val192. Examination of the equivalent extrafacial hinge residues in the representative Cluster 1 to 5 SWEETs found that the Tyr and Asp residues are conserved across all these microalgae and fungi SWEETs, while the Lys residue is conserved in just two of the six structures (CrSWEET1 and AmaSWEET3), and there is no equivalent Val residue in any of the structures (Figure 7). Molecular dynamic analysis for OsSWEET2b revealed both Tyr61 and Asp189 are crucial for substrate translocation through the protein (Selvam et al., 2019). Asp189 is thought to hydrogen-bond to both Tyr61 and Lys65, stabilising the IF conformation, and substitution of all three residues resulted in a significant loss of transport activity (Han et al., 2017). Both Tyr residues use polar, Van der Waals and hydrophobic interactions with the substrate as it moves through and exits the transport pathway in AtSWEET1, AtSWEET13, and OsSWEET2b (Han et al., 2017; Selvam et al., 2019). The conservation of these residues across the Cluster 1 to 5 SWEETs indicates that these algal and fungal SWEETs will share the alternating access model of sugar transport (Figure 1C) that has been described for many eukaryotic and prokaryotic SWEET representatives (Latorraca et al., 2017; Selvam et al., 2019). Overall, a smaller, more restricted binding pocket cavity than typically observed in higher plant SWEETs allows us to predict that these Chlorophyta SWEETs function as small hexose or pentose transporters, despite showing broad conservation with the substrate binding and translocation mechanism of higher plant SWEETs.

### Potential functions and applications of green algae SWEETs

Chlorophyta algae, such as *C. reinhardtii* are mainly found in aquatic environments where access to carbon is limited. These algae have evolved methods of carbon concentrating and fixation of inorganic carbon to acclimatise to nutrient-limited environments (Kono et al., 2020). The CrSWEET1 protein was previously identified in low CO_2_ inducible transcriptomic screens (and has also been named as LCI36) where it was found to be moderately induced under low CO_2_ conditions (Miura et al., 2004; Brueggeman et al., 2012). While an increase in glucose transport is unlikely to be directly involved in the carbon concentrating mechanism, the need to remobilise sugars within the cell may be a consequence of CO_2_ deprivation and inhibited photosynthesis. In *A. thaliana,* sucrose-transporting SWEET proteins contribute to adaptation to osmotic stress (Durand et al., 2018), although whether algae SWEETs are involved in responses to conditions such as osmotic stress remains to be determined. There is also some evidence that algal sugar transporters including SWEETs are routes for transferring sugars to a pathogenic or symbiotic host, as is the case with some higher plant SWEETs (Gupta et al., 2021). For example, sugar transporters are thought to play a key role in the symbiotic feeding between the alga *Micractinium conductrix* and a *Paramecium bursaria* host, and although SWEET genes have been identified in this alga, they have not yet been confirmed as the sugar efflux pathway for the host (Arriola et al., 2018). Microalgae SWEETs have also been indicated to facilitate sugar scavenging from a marine algal host (the dinoflagellate *Scrippsiella acuminata*) by the parasite *Amoebophyra* spp. (Decelle et al., 2021). Although no experimental validation has been carried out in this study, expression of CrSWEET1 to 4 was observed in studies investigating transcriptomic response to environmental stress (Bell et al., 2016; Wase et al., 2019). This raises further questions of the endogenous function of these SWEETs and is the subject of ongoing research.

SWEET proteins also have potential biotechnological applications. Podolsky et al. (2021) postulated how SWEET proteins can be utilised to enhance fermentation of digested lignocellulosic biomass. Consumption of xylose represents a bottleneck for many microbial cell factories as there is a shortage of yeast strains that can metabolise xylose; glucose uptake will often competitively inhibit xylose uptake. Specific SWEET proteins from *N. californiae* and *A. thaliana* (NcSWEET1 and AtSWEET7) have demonstrated the capacity to non-discriminatively co-transport glucose and xylose, allowing for efficient fermentation of digested lignocellulosic biomass using genetically modified yeast expressing the protein (Kuanyshev et al., 2021; Podolsky et al., 2021). The present study has demonstrated how algal SWEET proteins may have a binding site more suited to the selection and transport of pentose sugars (Figure 6) and are closely related phylogenetically to NcSWEET1 (Figure 2). Bioprospecting for pentose- and hexose-utilising SWEETs, such as from algae species, could yield credible candidates for engineering novel fermentative strains of yeast. Furthermore, as some plasma membrane-localised SWEET proteins have demonstrated the capacity for glucose efflux (Chen et al., 2010; Shammai et al., 2018), particularly when overexpressed, direct engineering of algal SWEETs may yield new outlooks for novel bio-refineries. For example, over-expression of algal SWEET genes may allow the controlled efflux of glucose for use as a bioethanol feedstock, without the need for harsh, energy-intensive or expensive methods of cell pre-treatment and hydrolysis to release the sugars.

There are key questions that remain outstanding following this *in silico* work which require subsequent experimental validation. These include confirmation of sub-cellular localisation for specific SWEETs and experimental determination of their substrate, as well as the kinetics and mode of regulation of sugar transport. Additionally, to date, significant investigation has focused on the diverse functional role of SWEET proteins across plant lineages, including their involvement in sugar partitioning, seed development or symbiotic relationships (Gao et al., 2018; Jeena et al., 2019; Gupta et al., 2021; Xue et al., 2022). This has greatly improved our understanding of carbon flux and transport in plants; however, the role of sugar transporters in microalgae is much less well understood. The present study should lead to more investigation into the functional role of SWEET sugar transporters in carbon partitioning within individual algal cells.

### Conclusion

SWEET proteins have been identified in all kingdoms of life, including in different classes of algae (Jia et al., 2017), but to our knowledge, this is the first study to perform a focused bioinformatics investigation of the SWEET protein family across the Chlorophyta taxon of algae and carry out an initial phylogenetic and structural characterisation of representative sequences. Sub-cellular localisation predictions showed possible diversity in functionality between SWEETs of different clusters. Although we have shown that Chlorophyta SWEETs, alongside selected fungal SWEETs are phylogenetically distinct from Streptophyta SWEETs, there is phylogenetic diversity within the Chlorophyta-fungal clade. Structural modelling of selected algal and fungal SWEETs, distinguished from five clusters display clear conservation of most of the key amino acid residues that are known to determine substrate specificity and transport mechanisms in higher plant and bacterial SWEETs. However, there is some divergence of binding site residues due to different side chain characteristics that would be predicted to give rise to differences in the sugar transport characteristics of distinct algal SWEETs, and differences in other structural features such as length of the C-terminal tail. Therefore we have shown that a degree of structure–function variation exists between each cluster, thus demonstrating potential functional diversity that correlates with the Chlorophyta-fungal SWEET evolutionary model.

Specifically, Cluster 1 proteins, which are composed exclusively of Chlamydomonadales-derived SWEETs, and predicted to be nearly all plasma membrane localised, have more substantial differences in THB structure with poor homology to SemiSWEET THB1 indicating possible evolutionary divergence. These SWEETs typically have a longer cytosolic C-terminal tail indicating different regulation modes including oligomerisation, have conserved Trp-Asn pairs, Pro tetrad, Tyr and Asp extrafacial gate residues, and show the presence of Met and Tyr binding pocket residues, indicating small sugar substrate selection.

Cluster 2 SWEET proteins have similar characteristics to those of Cluster 1 except that more members have possible vacuolar localisation and therefore some different cellular function. They mostly do not have a long C-terminal tail, but the THB1 domain does have stronger homology to SemiSWEET THB. Equivalent characteristics of binding pocket residues to those of Cluster 1 proteins also indicate the ability to bind and transport small sugars.

Cluster 3 SWEETs have a broader taxonomic composition as they include proteins derived from Chlamydomonadales- and Sphaeropleales species. Localisation to the vacuole is likely in five of the 14 SWEETs and only two of the Cluster 3 SWEETs have confident PM-localisation. As for the Cluster 2 SWEETs, they lack a long C-terminal domain and there is greater mismatching of residues across the proteins, particularly for the Sphaeropleales-derived SWEETs, potentially indicating some functional variation that is yet to be evaluated. Extrafacial gate and binding pocket residues are conserved in the same manner as for the Cluster 1 and 2 SWEETs, showing that residues that are seen as deterministic for substrate selection (whether monosaccharide or disaccharide sugars) were conserved among Chlorophyta SWEETs, although divergent from higher plant SWEETs.

Cluster 4 SWEETs derived from *N. californiae* fungi also consistently lack a long C-terminal tail, and also have homology to SemiSWEET THB structure. There is some variation in the Pro tetrad that makes up the extrafacial gate region, and the presence of an Ile residue in the binding pocket is predicted to allow pentose sugar selectivity.

Finally, Cluster 5 composed of *A. macrogynus*-derived SWEETs has equivalent conservation of the substrate binding and translocation mechanism residues compared to the algal SWEETs, but the presence or absence of a long C-terminal tail is not conserved across the cluster.

## Data availability statement

The datasets presented in this study can be found in online repositories. The names of the repository/repositories and accession number(s) can be found in the article/Supplementary material.

## Author contributions

JF, MA, and JP conceived and designed the data analysis and reviewed and edited the manuscript. JF and MA performed data analysis. All authors contributed to the article and approved the submitted version.

## Funding

JF was supported by a BBSRC DTP PhD studentship (grant number BB/M011208/1).

## Conflict of interest

The authors declare that the research was conducted in the absence of any commercial or financial relationships that could be construed as a potential conflict of interest.

## Publisher’s note

All claims expressed in this article are solely those of the authors and do not necessarily represent those of their affiliated organizations, or those of the publisher, the editors and the reviewers. Any product that may be evaluated in this article, or claim that may be made by its manufacturer, is not guaranteed or endorsed by the publisher.

## Footnotes

^1^ http://tree.bio.ed.ac.uk/software/figtree
