# Supplementary material for: Phylogenetic analysis and structural prediction reveal the potential functional diversity between green algae SWEET transporters
Source: Front Plant Sci. 2022 Sep 15;13:960133. doi: 10.3389/fpls.2022.960133 (PMC9520054; doi:10.3389/fpls.2022.960133)
Supplement: Supplementary file 2 [file Data_Sheet_1.PDF]

Supplementary Figures

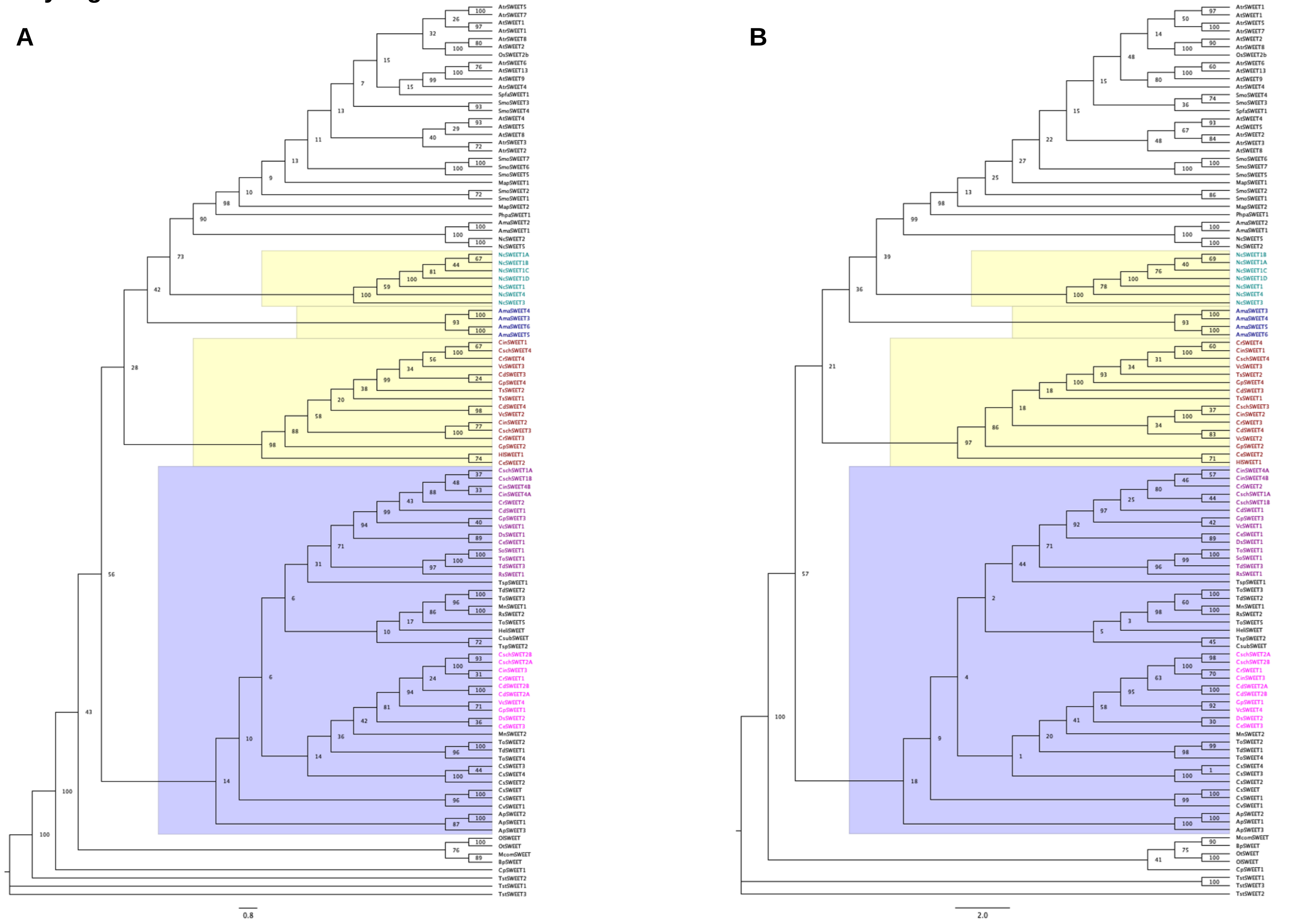

Supplementary Figure 1. Phylogenetic cladogram trees of selected SWEET protein sequences from representative algae and fungi, and outlier plant sequences. The maximum-likelihood trees were generated using either the BLOSUM62 (**A**) or LG (**B**) substitution matrix. Bootstrap replication values are shown at each node. The highlighted sequences in blue and yellow shading depict the two sub-clades of algae and algae-fungi SWEETs within the larger algae-fungi clade, as determined by the bootstrap probability. Algae and fungi SWEETs that form distinct clusters based on CLANS analysis, as well as the plant outlier SWEETs are labelled in colour. The branch length scale bar indicates the evolutionary distance of amino acid substitutions per site.

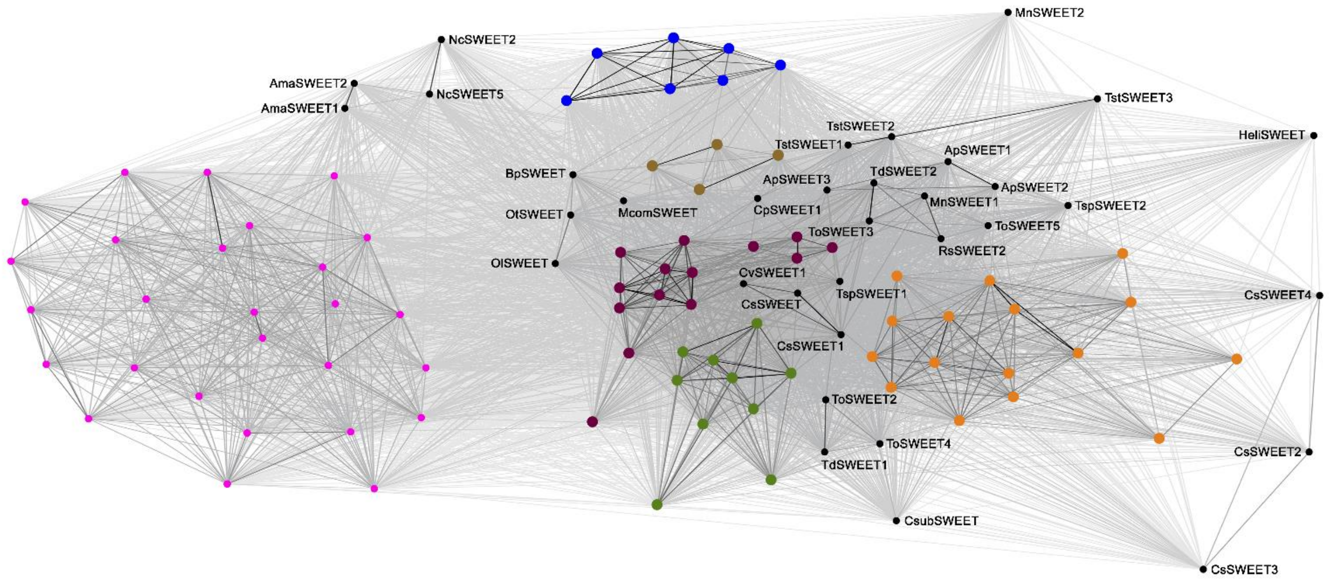

Supplementary Figure 2. A two-dimensional cluster analysis of the SWEET sequences performed using CLANS highlighting the ‘unclustered’ proteins (labelled). Each symbol represents one of the SWEET proteins and is coloured on the basis of the phylogenetic groups shown in Figure 2. Grey lines represent protein connections with reciprocal BLAST hits at $P < 10^{-5}$.

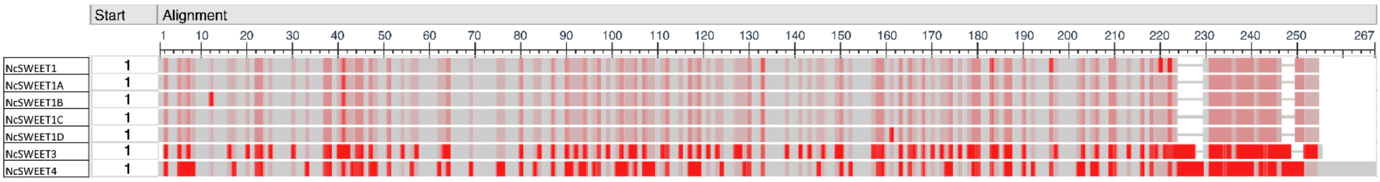

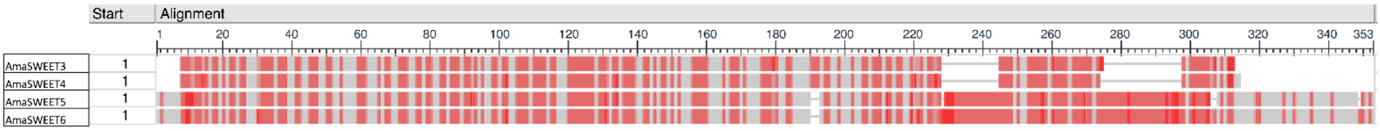

Supplementary Figure 3. COBALT multiple sequence alignment of Cluster 4 (NcSWEET) and Cluster 5 (AmaSWEET) fungi SWEET protein sequences. Amino acid conservation was scored based on frequency within each amino acid position (column). Grey shading indicates an identical residue at that position and darker shades of red indicate differences from residues in other rows in the alignment at that position.

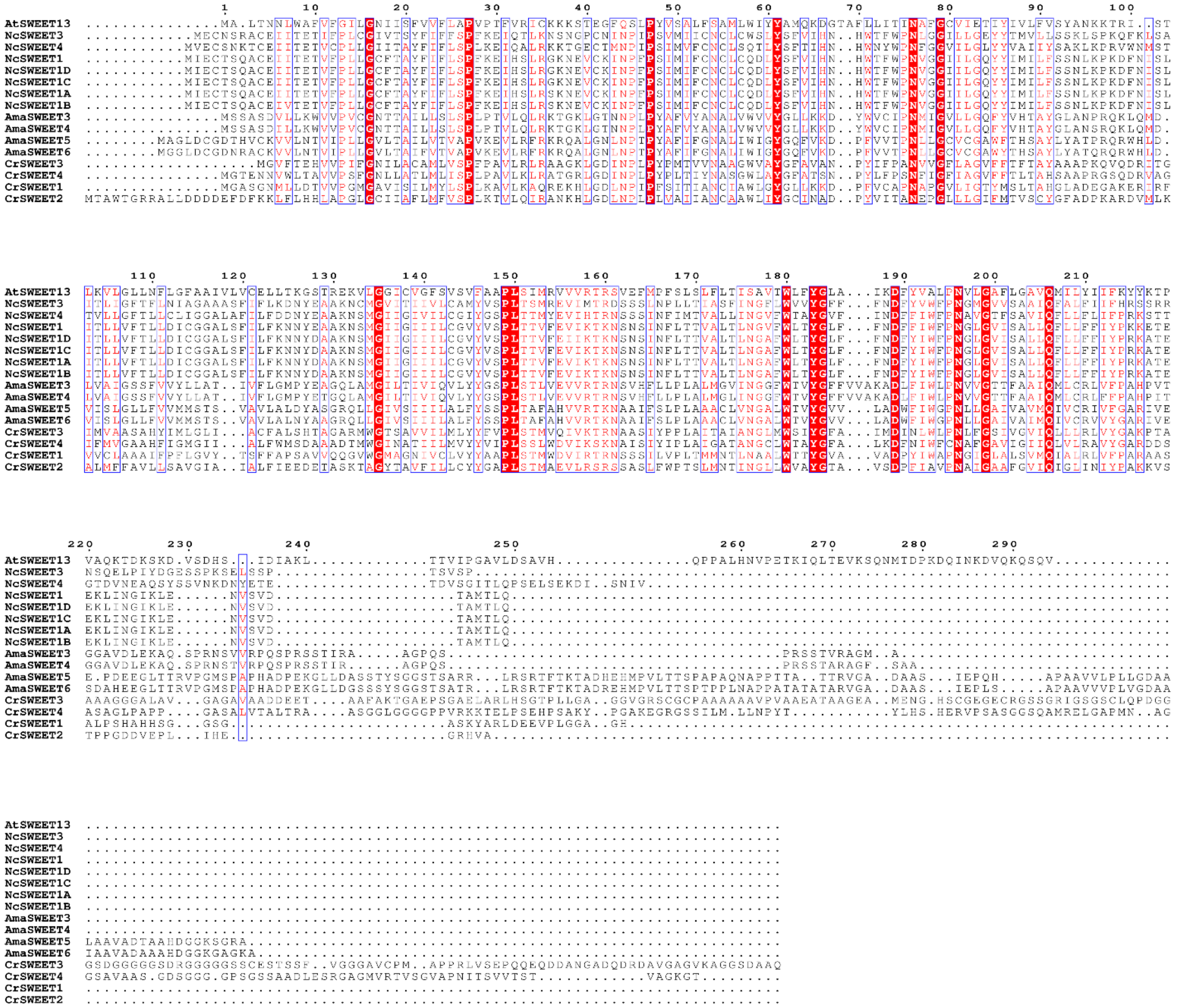

Supplementary Figure 4. Multiple amino acid sequence alignment of AtSWEET13 compared to *Chlamydomonas reinhardtii* SWEET proteins, Cluster 4 (NcSWEET) and Cluster 5 (AmaSWEET) proteins. Amino acids that are identical or similar are shown in red shading or red font, respectively. The numbering is based on the AtSWEET13 sequence.

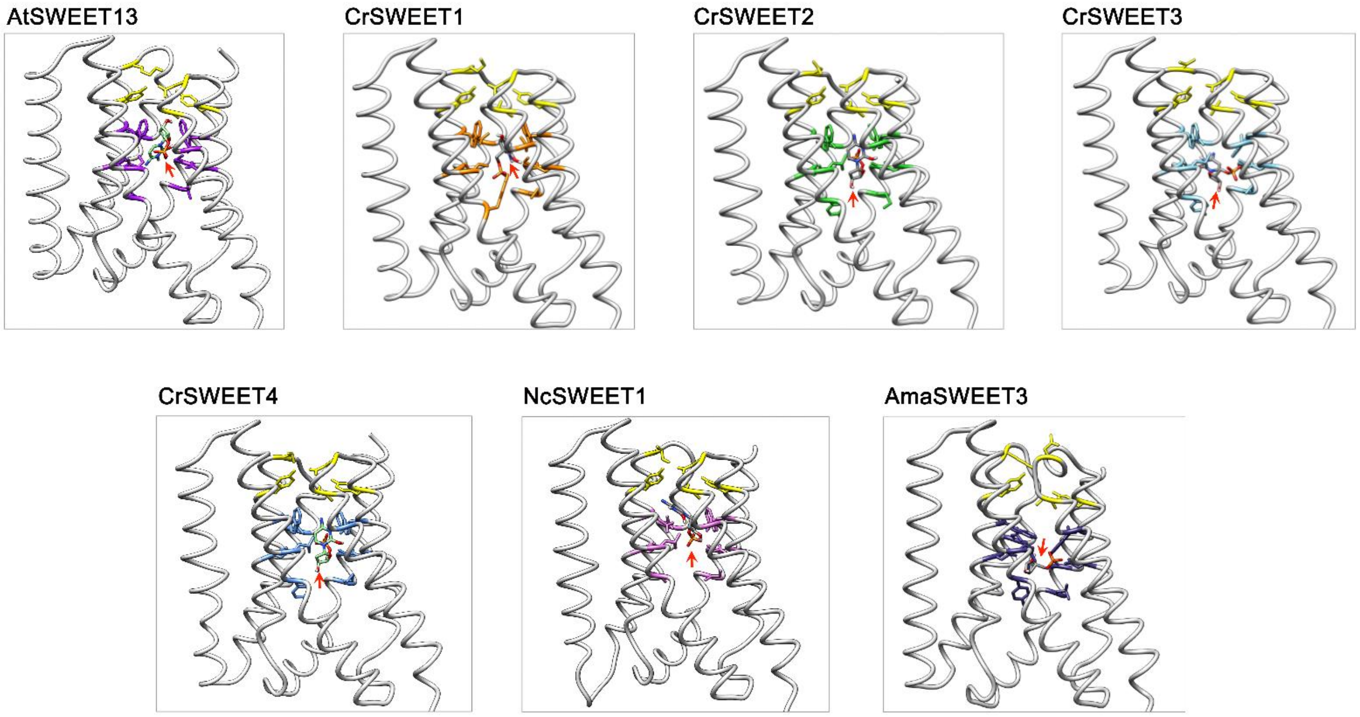

Supplementary Figure 5. Substrate docking simulations of AtSWEET13, CrSWEET1 to 4, NcSWEET1, and AmaSWEET3 (representatives of Cluster 1 – 5) determined using an AtSWEET13 template. Residues shaded in yellow are proposed to function in substrate interaction at the extracellular gate, while residues shaded in other colours are proposed to function in substrate interaction within the binding pocket. The DCM substrate that simulates glucose binding is indicated by a red arrow.
